# Using protein engineering to understand and modulate aggregation

**DOI:** 10.1016/j.sbi.2020.01.005

**Published:** 2020-02

**Authors:** Jessica S Ebo, Nicolas Guthertz, Sheena E Radford, David J Brockwell

**Affiliations:** 1 Astbury Centre for Structural Molecular Biology, University of Leeds, Leeds, LS2 9JT, UK; 2 School of Molecular and Cellular Biology, Faculty of Biological Sciences, University of Leeds, Leeds, LS2 9JT, UK

## Abstract

Protein aggregation occurs through a variety of mechanisms, initiated by the unfolded, non-native, or even the native state itself. Understanding the molecular mechanisms of protein aggregation is challenging, given the array of competing interactions that control solubility, stability, cooperativity and aggregation propensity. An array of methods have been developed to interrogate protein aggregation, spanning computational algorithms able to identify aggregation-prone regions, to deep mutational scanning to define the entire mutational landscape of a protein’s sequence. Here, we review recent advances in this exciting and emerging field, focussing on protein engineering approaches that, together with improved computational methods, hold promise to predict and control protein aggregation linked to human disease, as well as facilitating the manufacture of protein-based therapeutics.

## Highlights

• Computational methods identify aggregation-prone regions in protein sequences as a guide for protein engineering.
• Rational methods can reveal genotype-phenotype relationships and to introduce reactive handles for many applications.
• Directed evolution allows the identification of aggregation-resistant proteins in the absence of mechanistic understanding.
• Deep mutational scanning can potentially reveal the effect of every amino acid at every residue on protein aggregation.

## Graphical abstract

**Figure:**
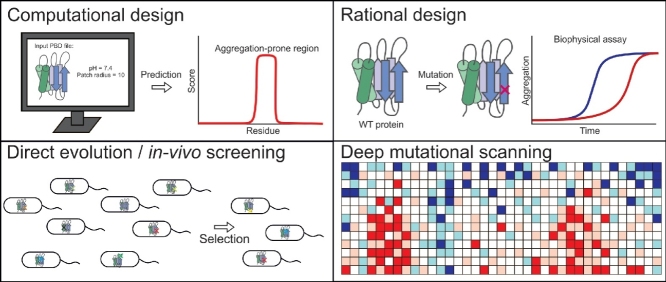

**Current Opinion in Structural Biology** 2020, **60**:157–166

This review comes from a themed issue on **Proteins**

Edited by **Jan Steyaert** and **Todd O Yeates**

For a complete overview see the Issue and the Editorial

Available online 19th February 2020

https://doi.org/10.1016/j.sbi.2020.01.005

0959-440X/© 2020 The Authors. Published by Elsevier Ltd. This is an open access article under the CC BY license (http://creativecommons.org/licenses/by/4.0/).

## Introduction

**Figure 1:**
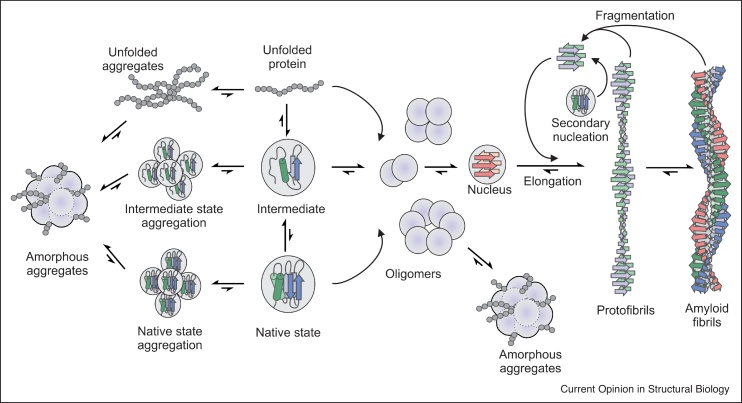
Schematic illustration of aggregation pathways. The precursor of aggregation may be the unfolded, partially folded or native state of a protein. During amyloid formation, oligomeric species formed from the initial aggregation-prone monomer, can then assemble further to form higher-order oligomers, one or more of which can form a nucleus, which, by rapidly recruiting other monomers, can nucleate assembly into protofibrils and amyloid fibrils. As fibrils grow, they can fragment, yielding more fibril ends that are capable of elongation by the addition of new aggregation-prone species [86]. Alternatively, amorphous aggregation can occur via one or more aggregation-prone species growing into larger species, by Ostwald ripening or other self-association mechanisms [87].

It has been long been recognised that protein aggregation pervades human morbidity and mortality [1] and impinges on our ability to produce life-saving and life-changing protein therapeutics both rapidly and economically [2]. It is now widely understood that as well as adopting soluble, functional structures, many proteins can also self-assemble forming structured aggregates such as amyloid fibrils [3,4], or to undergo liquid-liquid phase-separation [5,6]. The later process drives the formation of membraneless organelles that can be functional (such as in the nucleolus [7]), or causative of cellular dysfunction and disease (such as in virus replication [8] or in protein aggregation disorders [9]) (Figure 1). The ability of proteins to catalyse reactions, to form stable scaffolds, and to bind ligands tightly and with high specificity, has enormous potentials for the use of proteins in industry [10,11]. However, a major challenge in the use of proteins for such applications lies in their instability, conformational dynamics and inherent tendency to aggregate. There is thus an important and currently unmet need to be able to identify protein sequences that may have undesired properties and to engineer their sequences to improve their properties.

While aggregation-prone regions (APRs) can be readily identified in short peptide segments using computer algorithms [12, 13, 14, 15], for intrinsically disordered proteins (IDPs) and globular proteins it is still difficult, if not impossible, to identify aggregation-prone and aggregation-resistant sequences under a given set of conditions. This is because aggregation (taken here to be any non-native oligomeric state) can proceed through diverse mechanisms, driven by distinct physico-chemical mechanisms (Figure 1). In addition, the observed aggregation propensity of each protein sequence/structure on each pathway results from a complex convolution of the effects of its sequence on thermodynamic stability, structure, cooperativity and dynamics, which all also depend on the solution conditions (pH, temperature, ionic strength, solvent, nature of surfaces, etc.). For each and all of the pathways traversed, detailed understanding of the molecular mechanisms of the early stages of aggregation remain elusive. By linking changes in sequence to changes in biophysical and cellular behaviour, powerful new approaches in protein engineering are now able to provide a wealth of insight into this process, which can then be used to enhance the performance of computer algorithms so they are better able to predict protein behaviour. Here we discuss how the integration of protein engineering approaches with orthogonal methods including computational and high-throughput phenotypic screening methods, is now set to tackle this difficult problem.

## Delineating aggregation mechanisms using rational protein engineering methods

**Figure 2:**
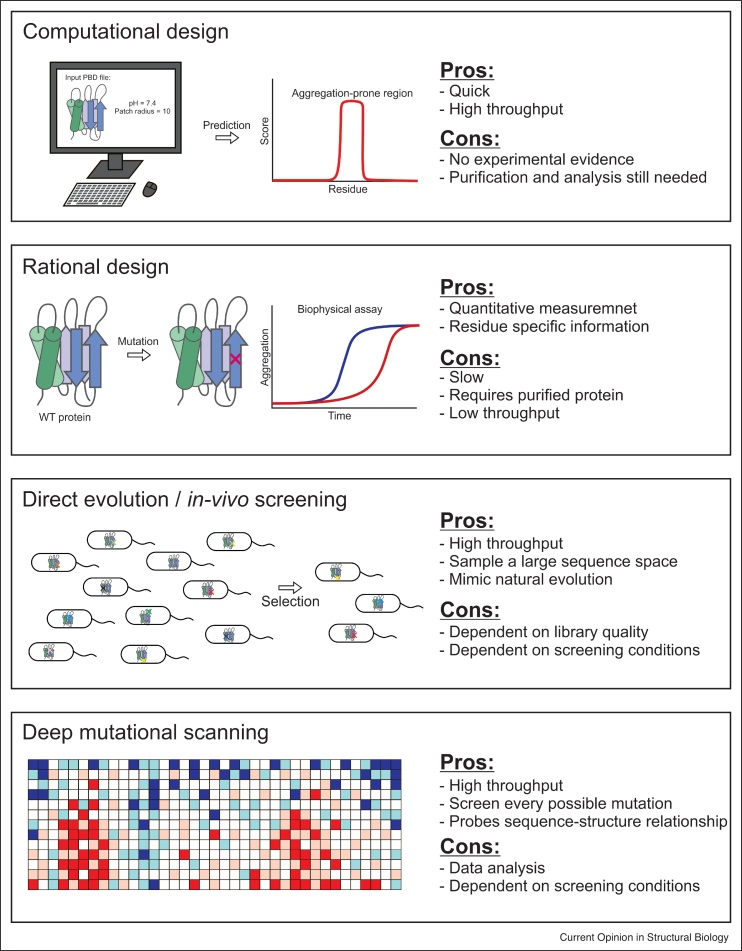
Summary of different methods for measuring and predicting protein aggregation. Computational methods can predict aggregation-prone regions using sequence or structure input. Rational design involves introducing specific mutations into a protein and subsequent analysis of the mutational effect in comparison to the behaviour of the wild-type protein. Directed evolution and in vivo screening methods obviate protein purification and large numbers of variants can be screened to identify proteins with enhanced properties. Finally, deep mutational scanning can potentially samples every possible mutation and enables quantification of the effect on protein stability or aggregation to be determined in vivo.

Rational redesign (i.e. the substitution of a small number of residues in a protein sequence with those having the desired physico-chemical or spatial properties) is an attractive approach to modulate protein aggregation when there is prior knowledge of the mechanism of aggregation (Figure 2) (e.g. by altering a protein–protein interface required for aggregation [16, 17, 18]). Approaches such as alanine scanning can also be used to identify or confirm predictions of residues key to the control of aggregation [19,20]. The ability to identify ‘aggregation hotspots’ has been facilitated by the development of at least 40 different algorithms [12, 13, 14, 15]. While differing in their metrics, these programs generally consider three characteristics which control protein aggregation: solubility, thermodynamic stability and aggregation propensity. These computational tools, summarised in Table 1, provide powerful information with which to start any study of protein aggregation by portraying the inherent aggregation propensity of the protein sequence. However, some consider local protein sequences (generally 4-6 residues in length), leaving open the important questions of how this inherent insolubility/aggregation potential is realised in the context of the entire protein sequence, whether disordered (as in the unfolded state or for IDPs) or when ‘hidden’ by the native 3D structure of the protein.

**Table 1:** Computational methods to predict and modulate protein aggregation. Methods are grouped by calculated metric and are subdivided into methods that use primary or tertiary sequence data. Algorithms denoted with ‘P’ represent those specific to Prion formation.

Protein solubility	
Sequence	Structure
Aggrescan [88] CamSol intrinsic [89] Protein-Sol [90] Proso II [91]	Aggrescan3D 2.0 [39] CamSol [89] SAP [92] SOLart [93]

Aggregation propensity	
Sequence	Structure
Zyggregator [94] TANGO [22] Pafig [95] SALSA [96] WALTZ-DB 2.0 [97] AmyCo^P^ [98] pWALTZ^P^ [99] PrionW^P^ [100] PLAAC^P^ [101] pRANK^P^ [102] PAPA^P^ [103]	PASTA 2.0 [104] Solubis [42] FoldAmyloid [105] NetCSSP [106] BETASCAN [107] STICHER [108] Zipper DB [109] AmyloidMutants [110] AMYLPRED2 [111]

## Detecting aggregation-prone regions in primary sequences

More than 80 % of proteins possess at least one region in their sequence that has a propensity to aggregate (i.e. APRs [21]), calculated based on hydrophobicity, charge patterning, aromatic content and β-sheet propensity [12]. These algorithms use the primary amino acid sequence to predict APRs via empirical training sets or/and calculation based in the known physicochemical properties of the 20 canonical amino acids [12]. One of these algorithms, TANGO [22] (Table 1), identifies APRs by calculating the propensity of penta-peptide sequences to form buried β-sheets, using an algorithm trained on experimental measurement. In an exciting recent application of this algorithm, Khodaparast *et al.* [23^•^] identified APRs enriched in the *Escherichia coli* proteome, and used the resulting information to develop new antibacterial agents by expression of redundant APRs (that were not sequence unique in the genome). Expression of 125 of these sequences resulted in cell death by inducing widespread aggregation of 541 proteins (identified using mass spectrometry) into cross β-structure-enriched inclusion bodies. In marked contrast, overexpression of unique APR sequences within the proteome had no effect. Antimicrobial amyloid-nucleating peptides were bactericidal for a large number of Gram-negative bacteria, suggesting that the approach may have therapeutic potential. Similar approaches from the same groups have also been used as anti-cancer strategies [24], suggesting the general utility of this method to exploit protein aggregation for beneficial purposes.

## Effect of ‘order’ in intrinsically disordered proteins

Transient structure formed within IDPs and short peptides can profoundly affect the observed aggregation rate of APRs. For example, the aggregation of Tau, a largely unstructured 441-residue protein which is associated with several neurodegenerative diseases, including Alzheimer’s, Pick and chronic traumatic encephalopathy [25, 26, 27], is thought to be largely driven by the amyloidogenic six-residue peptide sequence ^306^VQIVYK^311^ [28^••^]. Perplexingly, mutations genetically linked to tauopathies such as P301L/S are found outside this sequence. This is similar to the positional relationship between point variants of α-synuclein associated with early onset familial Parkinson’s disease and the non-amyloid component (NAC) region shown to be necessary and sufficient for aggregation [29,30]. Cross-linking studies, together with molecular dynamics (MD) simulations, showed that residues 295-311 of Tau form a β-hairpin, sequestering the APR, and slowing aggregation. Accordingly, destabilising the β-hairpin (by substitution of P301 with a bulky leucine residue) was to found to speed up aggregation, while stabilising it (via adding a Trp-zip motif to the termini of the β-hairpin, in the P301L background) slowed down aggregation [28^••^]. These elegant protein engineering experiments were thus able to confirm β-hairpin formation as a controlling mechanism of aggregation, in which the aggregation potential of the APR is modulated by specific structure formation in a region that both flanks and overlaps with the APR.

The transient and often promiscuous intra-molecular and inter-molecular interactions that control aggregation of both IDPs and initially structured proteins are challenging to study, but are necessary to understand and map because of their central importance in initiating aggregation. As a consequence of their dynamic and heterogeneous nature, high resolution structural techniques to map these important protein–protein interactions (Figure 1) are difficult, if not impossible to perform. However, these sequences can be engineered to allow site-specific introduction of specific reagents or reporters to gain low resolution information. These include cross-linking reagents, such as diazirines [31^•^], ruthenium complexes (PICUP) [32] and disuccinimidyl suberate (DSS) [33], which when coupled with mass spectrometric techniques [34], allow identification of pairs of residues that are spatially localised within the dynamic ensemble, even if only transiently populated [35]. Other reagents allow spectroscopic analyses. For example, introduction of spin labels (introduced via unique Cys residues) at single sites across proteins allows identification of transient interactions between sequence-distant residues, or between protein molecules using NMR (using an approach known as Paramagnetic Relaxation Enhancement [36]) or EPR (using Pulsed electron double resonance (PELDOR)/double electron-electron resonance (DEER) EPR spectroscopy [37]. These methods have been applied to α-synuclein, revealing that this IDP makes extensive intra-molecular and inter-molecular contacts which are highly sensitive to environmental conditions [29,37]. Such properties are reminiscent of those described above for Tau, especially as early onset familial missense variants occur outside of the main amyloid core for both proteins, suggestive of similar mechanisms at work that control the aggregation of these IDPs *in vitro* and possibly also *in vivo*. Similarly to Tau, formation of a β-hairpin structure (residues 37 to 54) in a region upstream to NAC (residues 61-95) in α-synuclein, induced upon complexation with a β-wrapin engineered binding protein, resulted in inhibition of amyloid formation [38]. These methods often yield low resolution, relatively sparse structural information, but by integrating the outputs from different approaches, remarkably precise molecular mechanisms of aggregation can result, especially when complemented with MD simulations. For example, Bunce *et al.* [31^•^] used fluorescence quenching of an extrinsic fluorophore (TAMRA-Ahx) and cross-linking studies to determine how the peptide Aβ_16-22_ (a fragment of Aβ_40/42_ associated with Alzheimer’s disease) aggregates and is able to catalyse self-assembly of Aβ_40_ via secondary surface nucleation.

## Effect of ‘disorder’ in the aggregation of globular proteins

Understanding the effect of protein dynamics, sequence and solution conditions is also critically important for determining, and hence predicting, the potential of globular proteins to aggregate. Given that aggregation can occur from the native state, or from partially or globally unfolded species (Figure 1), our ability to predict aggregation requires understanding of the local and global unfolding properties of the protein and how this depends on sequence and solution conditions. Simulation (and quantification) of protein dynamics *in silico* offers a solution to this problem, but requires greater computational resources and, in some cases, development of force fields able to accurately simulate protein behaviour. Aggrescan3D 2.0 [39] (Table 1) addresses this issue by using CABS-flex [40,41] for rapid simulations (10 nanoseconds length) of near-native dynamics of globular proteins. This ‘dynamic mode’ of Aggrescan3D 2.0 yielded higher aggregation propensity estimates for 80 % of the proteins tested relative to the value obtained from static structures. An alternative approach is to integrate rapid computational methods to predict protein solubility with algorithms able to predict thermodynamic stability. Solubis [42,43] (Table 1), for example, combines TANGO [22] (to identify APRs) with FoldX [44] (to compute the effect of amino acid substitutions on thermodynamic stability). Solubis [42,43] can be used to identify positions in a protein structure able to accommodate gatekeeper residues (i.e. residues with low β-sheet propensity (e.g. Pro) and high solubility such as the charged amino acids (Arg, Lys, Glu and Asp) with minimal changes in protein stability (ΔG°_UN_). This allows the redesign of proteins to retain stable and native folds, but to reduce aggregation propensity. This approach has been used successfully to decrease the aggregation kinetics of the Protective Antigen protein from *B. anthracis* [42], a key component in Anthrax vaccines [45], while preserving the native structure and function. This highlights the power of utilising the interdependency of solubility, stability and aggregation propensity to determine and re-engineer a protein’s aggregation potential.

## Understanding the diverse effects of electrostatics on protein aggregation

Proteins containing low complexity prion-like domains (PRDs), typically IDPs enriched in glycine and hydrophilic residues, play an important role in the formation of liquid-liquid phase separated membrane-less organelles such as the nucleolus, stress granules and P-bodies [46], and may allow generation of selectable genetic variability akin to that previously reported for prions [47]. Despite the relative depletion of hydrophobic residues in PRDs, reversible amyloid fibril formation can occur upon liquid-liquid phase separation of such sequences, and hence these sequences are known as LARKS (low-complexity aromatic-rich kinked segments) [48]. One such example is hnRNPA1 [49^••^], an RNA binding protein in which missense mutations are associated with neurodegenerative diseases [50]. Scanning the low complexity (LC) domains for segments containing (Asn)-Asp-(Asn) and (Gly)-Phe/Tyr-(Gly) motifs identified three peptides which formed a hydrogel composed of amyloid fibrils that dissociated upon an increase in temperature [49^••^]. The structure of the first reversible amyloid core (termed hnRAC1) revealed the cross-β architecture expected for an amyloid fibril, but with notable differences, thought to be important for their function. Firstly, the inter-sheet interface was composed of hydrophilic Asn residues compared with the dry steric zipper typical of amyloid [3,4]. The fibre was further destabilised by the stacking of an aspartic acid (D214) along the exterior face of its parallel in-register β-sheets. Finally, the structure revealed a kink at G211, thought to allow hydrogel formation by sterically facilitating inter-fibrillar cross-linking via π-π stacking of the adjacent Phe and other residues. Accordingly, an hnRAC1 peptide containing G211V, or F210A, or F216A displayed reversible fibril formation, but impaired hydrogel formation. Conversely substituting the destabilising aspartic acid residue (D214V/N) in hnRAC1 resulted in irreversible fibril formation. Interestingly, Asp, Val or Asn substitutions are also found in familial amyotrophic lateral sclerosis (ALS) patients and result in irreversible fibril formation [51]. Taken together, the results provide a structural rationale for ‘maturation’ of irreversible amyloid fibrils within liquid-liquid phase separated low complexity PRDs. The recognition that the aggregation propensity (and liquid-liquid de-mixing) of PRDs is driven by sequences that are chemically and sterically distinct to those involved in amyloid formation [52] has led to the development of AMYCO [53] an algorithm specialised for the prediction of PRDs (Table 1).

Electrostatic interactions are also important drivers and modulators of the aggregation of globular proteins, with pH and ionic strength being important determinants of aggregation both by increasing the probability of proteins unfolding, and by changing the probability of productive protein–protein interactions between transiently exposed APRs in non-native states [54, 55, 56]. The aggregation of natively structured proteins can also be problematic for proteins produced at scale, such as in the biopharmaceutical industry in which proteins are manufactured in high volume and at high concentration [2]. In these cases, aggregation is reversible (at least in the initial stages) and is driven by intermolecular contacts mediated by the presence of hydrophobic or charge-complemented patches on the protein surface, via a mechanism referred to as ‘colloidal aggregation’ [57] (Figure 1). ‘Supercharging’ proteins by introduction of an excess of acidic or basic residues throughout the protein [58, 59, 60] has been shown to reduce aggregation induced by such pathways. Alternatively, introducing defined clusters of specific charged residues that enhance protein stability and reduce protein–protein interactions has been shown to be an effective strategy to reduce aggregation [59,61, 62, 63].

## Using directed evolution and *in vivo* screening to define aggregation landscapes

Directed evolution (DE) methods involve generating diversity in the gene of interest and then isolating variants with improved characteristics from this library using phenotypic selection [64] (Figure 2). DE approaches have been used to develop aggregation-resistant biopharmaceuticals by screening for thermal resistance [65,66], or by utilising three selection methods (temperature, reduction and hydrophobicity) in parallel [67]. A potential disadvantage of optimising a protein’s sequence in this manner is that function is ignored, which can result in proteins with enhanced biophysical properties, but reduced activity, akin to sequence-stability trade-offs [68]. To counter this, Wang *et al.* described a soluble expression phage assisted continuous evolution method (SE-PACE) [69^•^]. Here, the protein of interest (POI) is linked in-frame to the N-terminal fragment of a split T7 RNA polymerase (to select for soluble POIs), as well as to the omega subunit of RNA polymerase (RNAP) to select for POIs with high target binding affinity using a bacterial two hybrid approach (using 434 phage cI repressor as the DNA-binding domain). Linking expression of soluble and functional POI to these distinct polymerases allowed both traits to be selected for simultaneously by only allowing expression of the minor coat protein III (pIII) required for progeny phage upon expression and complementation of both the N-terminal and C-terminal fragments of an intein transcribed by RNAP and T7 polymerase, respectively. Using this approach, a fivefold enhancement of expression, but unchanged target affinity was achieved for single-chain antibody fragments (scFvs), as well as enhancement of both expression and activity for the enzyme cytidine deaminase.

If aggregation occurs via a partially folded protein structure, the propensity to aggregate may not always correlate with protein thermal stability. For such proteins, it is necessary to develop alternative screens to create proteins with enhanced solution behaviour. One route to achieve this has recently been developed, in which an *E. coli* β-lactamase folding reporter links the innate ability of a protein to aggregate to antibiotic sensitivity by fusing the POI between two domains of β-lactamase [70,71]. The system has been shown to be able to differentiate between aggregation-prone and aggregation-resistant variants of diverse protein sequences and structures, including the aggregation-prone peptides Aβ_1-42_ and amylin, the aggregation-prone protein β_2_ microglobulin, and single domain antibodies. The system has also been used to screen for small molecule inhibitors of protein aggregation in the periplasm of *E. coli* [71] and for the selection of excipients able to suppress aggregation [72]. Other screens that link survival to dihydrofolate reductase (DHFR) activity have also been developed and used to identify peptide inhibitors of α-synuclein aggregation [73] and to characterise the phenotypes of 99 % of all the possible single-site substitutions of Aβ_1-42_ (see below) [74].

Screening peptides *in vivo* has also been used to gain deeper insight into the pathways by which toxic aggregates are formed [75^•^]. A combinatorial library of >10 million short cyclic peptides (S/T/C-X_1_-X_n_, where X is any amino-acid and n = 3-5) was produced in bacteria using split intein-mediated circular ligation of peptides and proteins [76]. The peptides were then screened for their ability to reduce aggregation monitored by a reduction in fluorescence of an Aβ_1-42_-GFP fusion reporter by fluorescence-activated cell sorting [75^•^]. Biochemical analysis of clones that increased fluorescence revealed penta-peptides that halt Aβ_1-42_ aggregation by stabilising β-sheet-like structures. These peptides also reduced toxicity measured in primary neuronal cell lines and *in vivo*.

## Developing enhanced understanding of protein behaviour using deep mutational scanning

Deep mutational scanning (DMS) can be used to reveal the effect of thousands of different single amino-acid substitutions on a protein’s properties by quantifying the relative change of abundance of each member of the library under a suitable selective pressure using next generation sequencing methods [77]. This approach is extremely powerful as it combines the strengths of both ‘traditional’ protein engineering methods (quantifiable sequence-phenotype relationships) and DE methods (the ability search vast areas of sequence space without protein purification) (Figure 2). DMS has thus found broad application from structure determination [78,79] to developing a better understanding of the determinants of protein thermodynamic stability [80] and even the utility of alanine scanning [81].

Two studies have recently used DMS to gain a broader understanding of the relationship between sequence and aggregation mechanism. Firstly, to investigate the molecular determinants of Aβ_1-42_ aggregation Gray *et al.*, [74], used selective growth pressure in yeast cells, by fusing Aβ_1-42_ to DHFR and growing a library of Aβ_1-42_ variants using methotrexate as a selective pressure for DHFR function. This screen evaluated 791/798 of all possible single amino acid substitutions of Aβ_1-42_. Remarkably, 25 % of the variants were more soluble than Aβ_1-42_, with the others showing unchanged or increased aggregation propensity. Substitutions to Asp and Pro enhanced solubility the most (presumably by increasing charge or decreasing β-strand propensity, respectively), whereas substitutions with Trp or Phe were associated with greater aggregation (presumably by increasing hydrophobicity). This mutational information revealed residues 17-20, 31-32, 34-35, 39 and 41 as ‘hotspots’ important for Aβ_1-42_ aggregation, which most likely form buried β-strands. Interestingly, these concur with predictions of APRs, for example, using TANGO and Zyggregator [82]. In the second example, Bolognesi *et al.* exploited the ability to measure the sequence-function relationships of thousands of variants in parallel to understand the relationship between aggregation and toxicity focussing on the PRD of the TAR DNA-binding domain 43 (TDP-43), the aggregation of which is linked to ALS [83^••^]. Comparison of the relative change in the population of >50 000 variants of yeast cells containing one or two substitutions in TDP-43 before and after induction revealed a 31 residue ‘toxic hotspot’, which correlates with the region of the protein in which mutations occur in ALS patients. Surprisingly, substitutions in this hotspot that increase hydrophobicity decreased toxicity, whereas substitutions that increase charge or polarity increased toxicity. Variants with increased hydrophobicity produced larger, stable aggregates that are less toxic than the small liquid-like loci found at the nuclear periphery for the more toxic variants. Furthermore, epistatic analysis of variants containing two substitutions suggested the presence of secondary structure in this apparently disordered domain. This powerful method can thus be used to identify the structural properties of IDPs *in vivo* and, further, to interrogate the relationship between the function and toxicity of amyloid versus protein assemblies in liquid-liquid phase separation.

## Future perspectives

The synergy between protein engineering and biophysical measurements *in vitro* with cellular approaches has been integral to developing our understanding of protein aggregation (Figure 2). The diversity of aggregates and aggregation mechanisms, together with the emerging realisation that even IDPs contain transient structure crucial to their function and aggregation potentials, and the finding that native state dynamics are crucial to understanding aggregation propensity, pose enormous current challenges to our ability to predict and modulate aggregation. The ability to rapidly survey the aggregation propensity of large numbers of highly homologous sequences using DMS methods together with statistical and machine learning methods, is now able to guide protein engineering [84,85] and, in the future, is sure to guide the development of new predictive algorithms. These large datasets, when integrated with detailed spectroscopic and cross-linking studies (all made possible by protein engineering approaches), MD simulations and cellular insights, will allow us in the future to define the relationship between sequence, structure, function and aggregation. This will allow genome engineering or the development of small molecules or biomolecules able to control protein aggregation and to develop and manufacture biotherapeutics more rapidly and economically. What is clear is that there is still much to learn, but the powers of modern protein engineering methods, combined with the ability to harness the information that results through machine learning, promises a step change into our ability to understand protein behaviour and to capitalise on the new knowledge to capture the complexity and powers of proteins for the benefits of humankind.

## CRediT author statement

JSE and NG prepared the original draft, SER and DJB reviewed and edited the manuscript.

## Conflict of interest statement

Nothing declared.

## References and recommended reading

Papers of particular interest, published within the period of review, have been highlighted as:

• of special interest
•• of outstanding interest
